# Xenogeneic-Free Human Intestinal Organoids for Assessing Intestinal Nutrient Absorption

**DOI:** 10.3390/nu14030438

**Published:** 2022-01-19

**Authors:** Makoto Inoue, Yuichi Tanaka, Sakiko Matsushita, Yuri Shimozaki, Hirohito Ayame, Hidenori Akutsu

**Affiliations:** 1 Research & Business Development Center, Converting Business Development Unit, Dai Nippon Printing Co., 250-1, Wakashiba, Kashiwa 277-0871, Chiba, Japan; inoue-m38@mail.dnp.co.jp (M.I.); Tanaka-Y64@mail.dnp.co.jp (Y.T.); Matsushita-S4@mail.dnp.co.jp (S.M.); ayame-h@mail.dnp.co.jp (H.A.); 2 Medical & Healthcare Division, Dai Nippon Printing Co., 1-1-1, Ichigaya-Kagacho, Shinjuku 162-8001, Tokyo, Japan; Shimozaki-Y@mail.dnp.co.jp; 3 Center for Regenerative Medicine, National Center for Child Health and Development (NCCHD), Department of Reproductive Medicine, Okura 2-10-1, Setagaya 157-8535, Tokyo, Japan

**Keywords:** intestinal organoid, intestinal absorption glucose, dipeptide, cholesterol, natural ingredient

## Abstract

Since many nutrients, including the three major ones of glucose, dipeptides, and cholesterol, are mainly absorbed in the small intestine, the assessment of their effects on intestinal tissue is important for the study of food absorption. However, cultured intestinal cell lines, such as Caco-2 cells, or animal models, which differ from normal human physiological conditions, are generally used for the evaluation of intestinal absorption and digestion. Therefore, it is necessary to develop an alternative in vitro method for more accurate analyses. In this study, we demonstrate inhibitory effects on nutrient absorption through nutrient transporters using three-dimensional xenogeneic-free human intestinal organoids (XF-HIOs), with characteristics of the human intestine, as we previously reported. We first show that the organoids absorbed glucose, dipeptide, and cholesterol in a transporter-dependent manner. Next, we examine the inhibitory effect of natural ingredients on the absorption of glucose and cholesterol. We reveal that glucose absorption was suppressed by epicatechin gallate or nobiletin, normally found in green tea catechin or citrus fruits, respectively. In comparison, cholesterol absorption was not inhibited by luteolin and quercetin, contained in some vegetables. Our findings highlight the usefulness of screening for the absorption of functional food substances using XF-HIOs.

## 1. Introduction

The small intestine is an organ that is involved many functions, such as absorption, digestion, metabolism, peristalsis, and immune responses [1,2,3,4]. Nutrients are mainly assimilated through transporters expressed on the brush border membrane of intestinal epithelial cells. Sodium/Glucose Cotransporter 1 (SGLT1) is a sodium ion gradient-dependent cotransporter and Glucose Transporter Type 2 (GLUT2) facilitates diffusion transport for the absorption of monosaccharide glucose. GLUT2 is also present in the intestinal basolateral membrane [5]. H+/oligopeptide cotransporter (PEPT1) absorbs amino acids and di/tripeptides [6,7]. With regard to cholesterol absorption, Niemann-Pick C-1-like-1 (NPC1L1) and diffusion transport pathways by micelle formation exist, but about 70% of cholesterol is absorbed via the former [8]. However, further details on transporters are required to more fully understand nutrient absorption in the small intestine.

More recently, it is thought that lifestyle-related diseases are prevented by the daily intake of healthy foods instead of medicines. Therefore, much attention has been focused on analyzing food ingredients with the aim of discovering new functional foods. For example, various beneficial ingredients contained in vegetables, fruits, and tea leaves are known to have potential for suppressing cancer risk, improving cardiovascular disease, and shutting down the absorption of sugars and lipids [9]. To evaluate the beneficial effects of functional food substances, studies using the colorectal cancer cell line, Caco-2, or animal models have been carried out. However, differences in the expression level of each transporter in tissue-derived cells from various cell types or species leave doubt about the application of the research findings from these models to humans [10,11]. Hence, an alternative method for the in vitro analysis of the effect of foods on diseases is required. Several groups have established organoids, which are self-organizing three-dimensional (3D) in vitro structures that have a cell composition and functionality similar to that of in vivo human organs [12]. Human intestinal organoids (HIOs) were differentiated from human pluripotent stem cells (hPSCs) using Matrigel; these were found to express intestinal markers and show dipeptide absorption activity [13]. However, such organoids showed their closed lumens with inward epithelial orientation are relatively inaccessible for absorption, distribution, and metabolism assays. By contrast, we have successfully developed an in vitro human intestinal model of xenogeneic-free human intestinal organoids (XF-HIOs) from hPSCs using micro-patterning substrates [14,15,16]. The XF-HIOs show the unique tissue and functional characteristics of human intestine: they are constructed from three germ–layered tissues, muscle cells, nerve cells as well as epithelial cells, and have external villi that are useful for the analysis of intestinal functions. In fact, we have analyzed the expression of metabolism-related genes and metabolism activity in XF-HIOs without requiring the dissociation of cells [17].

In the present study, we aim to assess nutrient absorption in XF-HIOs using fluorescent-labeled reagents, followed by elucidating the effect of well-known food functional ingredients that repress glucose and cholesterol absorption. Our findings suggest that XF-HIOs can be used as an in vitro model of nutrient absorption for functional food studies.

## 2. Materials and Methods

### 2.1. Cell Line and Media

Human-induced pluripotent stem cells (hiPSCs) were generated from a menstrual-blood-derived Edom cell line at National Center for Child Health and Development [18] and were cultured in StemFit AK02N (Ajinomoto, Tokyo, Japan) on vitronectin (VTN-N; Thermo Fisher Scientific, Waltham, MA, USA). The human colon cancer cell line, Caco-2, was obtained from the European Collection of Authenticated Cell Cultures (Cat# EC86010202, Lot 12F018) and cultured in Dulbecco’s Modified Eagle Medium (DMEM; Sigma-Aldrich, St. Louis, MI, USA) supplemented with 10% fetal bovine serum (FBS) and 1% penicillin/streptomycin (Thermo Fisher Scientific).

### 2.2. Quantitative Reverse Transcription PCR

Total RNA was extracted using RNeasy Plus Universal kits (Qiagen, Hilden, Germany), and first-strand complementary DNA (cDNA) was synthesized using SuperScript IV Mixture (Thermo Fisher Scientific), according to the manufacturers’ instructions. Three different healthy human adult small intestinal tissues (C1234226; BioChain Institute, Newark, CA, USA) were used as positive controls. Quantitative reverse transcription (RT)-PCR was performed in triplicate using a SYBR Green PCR Mastermix and QuantStudio 12 K Flex Real-Time PCR System (Thermo Fisher Scientific). Primer pairs used are shown in Table S1. GAPDH was used as an internal control. Data represent the mean ± standard error of the mean (S.E.) of three-to-nine independent experiments.

### 2.3. Immunofluorescence Staining

Organoids were embedded in IPGell (Genostaff, Tokyo, Japan), fixed in 4% paraformaldehyde, embedded in paraffin, and serially cut into 4 μm sections. Alternate sections were mounted on slides, deparaffinized, and antigen activated in Tris-EDTA buffer. After blocking in Protein Block Serum-Free (Dako, Glostrup, Denmark) at room temperature for 30 min, slides were incubated with primary antibody (Table S2) at 4 °C overnight, washed in Dulbecco’s phosphate-buffered saline (PBS, Thermo Fisher Scientific), and sequentially incubated with anti-mouse, anti-rabbit or anti-goat IgG antibody conjugated with Alexa Fluor 488 or Alexa Fluor 546 (Life Technologies, Carlsbad, CA, USA) at room temperature for 30 min. Cell nuclei were stained by treatment with 4′, 6-diamidino-2-phenylindole (DAPI; Biotium, Fremont, CA, USA). Cell fluorescence was analyzed using a BZ-X710 fluorescence microscope (Keyence, Osaka, Japan). 

### 2.4. Alcian Blue Stain

The organoids were embedded by IPGell (GENOSTAFF), fixed in 4% paraformalde- hyde, embedded in paraffin, and serially cut into 4 μm sections. For Alcian blue stain, sections were incubated in 1% Alcian blue in 3% acetic acid, pH 2.5, for 20 min and in 0.1% nuclear fast red for 2 min, dehydrated in ethanol, and cleared using xylene.

### 2.5. Barrier Function of the Organoids

The barrier function of organoids was tested using the permeability of the fluorescence marker, FITC-Dextran 4 kDa (FD-4; Sigma-Aldrich). Organoids were incubated in KnockOut DMEM (Thermo Fisher Scientific), with or without FD-4, at a final concentration of 0.01 mg/mL for 1 h. After treatment, the organoids were washed three times with Hanks’ balanced salt solution containing calcium chloride and magnesium sulfate (HBSS+), images photographed using a BZ-X710 fluorescence microscope, and the volume was measured using Dai Nippon Printing Co particle analytical software (Dai Nippon Printing Co., Tokyo, Japan) [19]. The organoids were homogenized in HBSS+, centrifuged at 10,000× *g* rpm for 5 min, and the fluorescence intensity of supernatants measured using a microplate reader (excitation at 485 nm, emission at 535 nm; BioTek Instruments, Winooski, VT, USA). In this study, bar graphs were calculated by (fluorescence intensity)/(volume of organoids).

### 2.6. Fluorescent Labeling Reagent Absorption Assay of the Organoids

Absorption assays were performed using a protocol on barrier function from each kit, according to the manufacturer’s instructions. Fluorophore-conjugated dipeptide β-Alanyl-N^ε^-(7-amino-4-methyl-2-oxo-2H-1-benzopyran-3-acetyl)-L-lysine (β-Ala-Lys-AMCA) (Tokyo Chemical Industry, Tokyo, Japan) absorption was performed using a previously described protocol [14]. The organoids were incubated in KnockOut DMEM containing β-Ala-Lys-AMCA, with or without captopril (Sigma-Aldrich), for 1 h at 37 °C. Fluorescence intensity was measured using a microplate reader (excitation at 355 nm, emission at 460 nm). Glucose absorption was measured using a 2-N-(7-Nitrobenz-2-oxa-1,3-diazol-4-yl) amino-2-deoxyglucose (2-NBDG) Glucose Uptake Assay kit (BioVision, Milpitas, CA, USA). The organoids were incubated in DMEM without glucose supplemented with 0.5% FBS containing 2-NBDG Glucose, with or without a compound such as phloretin, phlorizin, epicatechin-gallate (ECg), tangeretin, and nobiletin (Tokyo Chemical Industry) for 30 min at 37 °C. Fluorescence intensity was measured by microplate reader (excitation at 488 nm, emission at 520 nm). Cholesterol absorption was measured using a Cholesterol Uptake Assay kit (Cayman Chemical, Ann Arbor, MI, USA). The organoids were incubated in serum-free DMEM containing 7-nitrobenzo-2-oxa-1,3-diazole (NBD) Cholesterol, with or without a compound such as ezetimibe (Abcam, Cambridge, MA, USA), luteolin (Fujifilm Wako, Osaka, Japan), and quercetin (Tokyo Chemical Industry) for 24 h at 37 °C. We subsequently measured the fluorescence intensity using a microplate reader (excitation at 485 nm, emission at 535 nm).

### 2.7. ELISA for Muc2 in the Organoids

Secreted Muc2 was assessed using Human Muc2 ELISA Kit (Elabscience, TX, USA). The organoids were cultured for 48 h. After cultured, culture media were collected and centrifuged at 1000× *g* at 4 °C for 20 min, and supernatants were used for the kit, according to the manufacturer’s instructions. Culture media were used as negative controls.

### 2.8. Caco-2 Permeability Assay

The permeability of Caco-2 cells was assayed using 0.4 μm culture inserts (Corning, Corning, NY, USA. Briefly, 10^5^ cells/cm^2^ were suspended in DMEM medium with supplement and seeded into inserts in wells of a plate. These cells were then incubated for 21 days at 37 °C in 5% CO_2_. After incubation, we assessed the integrity of each Caco-2 monolayer by measuring its trans-epithelial electric resistance (TEER). The TEER was measured using a Millicell-ERS (Merck Millipore, Burlington, MA, USA). Permeability assays used TEER values greater than 500 Ω.cm^2^. Both apical and basolateral compartments of the culture inserts were washed with HBSS+. An aliquot of HBSS+ containing fluorescent-labeled dipeptide, glucose or cholesterol, with or without each inhibitor, was added to each apical compartment. After the reaction, HBSS+ in each basolateral compartment was collected and cells in each apical compartment were homogenized in HBSS+, centrifuged at 10,000× *g* rpm for 5 min, and supernatants assayed for fluorescence intensity using a microplate reader.

### 2.9. Statistical Analysis

We assessed statistical significance by comparing mean (± S.E.) values in a Student’s *t*-test for independent groups.

## 3. Results

### 3.1. Structure, Properties and Nutritional Absorption of XF-HIOs

**Figure 1:**
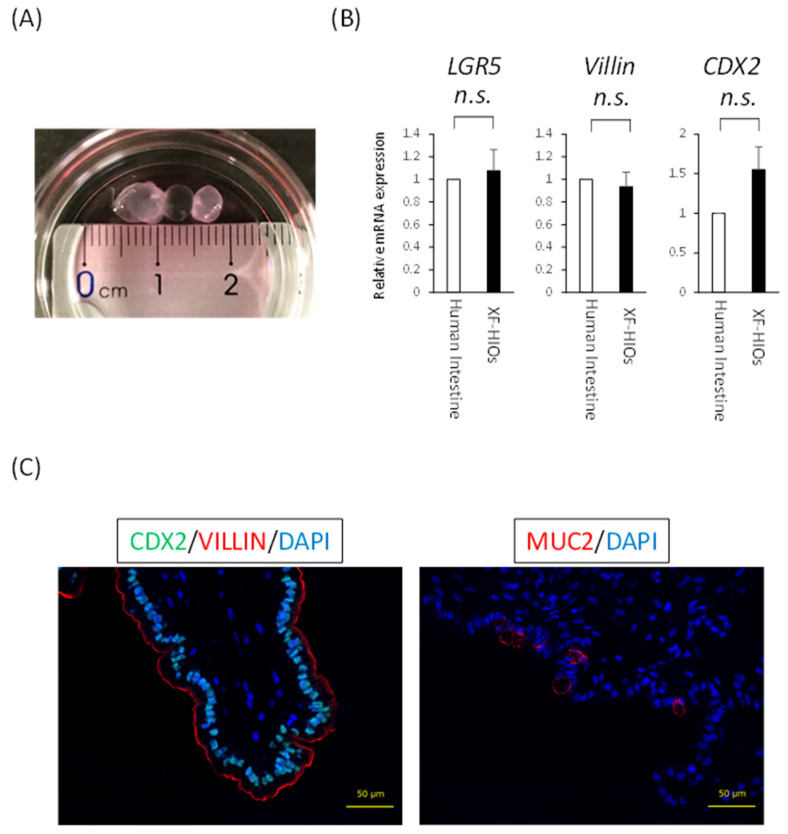
The structures and properties of XF-HIOs. (**A**) A photograph of three-dimensional xenogeneic-free human intestinal organoids (XF-HIOs). (**B**) The level of expression of mRNAs for *LGR5*, *Villin*, and *CDX2* relative to that of the human small intestine. Data shown represent the mean ± S.E. values of three independent experiments. n.s.; not significant. (**C**) Immunofluorescent images of XF-HIOs with anti-VILLIN, CDX2, and MUCIN2 (MUC2). DAPI, 4′, 6-diamidino-2-phenylindole. Scale bars: 50 μm.

Uchida et al. have previously reported the successful development of an in vitro intestinal organoid system under xenogeneic-free conditions using human embryonic stem cells or human-induced pluripotent stem cells (hiPSCs) [14]. We used the latter in this study. As for the structure and properties of XF-HIOs, these were spherical structures, about 1 to 10 mm in diameter, showing the mRNA expression of intestinal differentiation markers, such as LGR5, villin, and CDX2, at identical levels as those of the human small intestine (Figure 1A,B). As shown in Figure 1C, immunofluorescence staining for an intestinal transcription marker, CDX2, and a brush border cytoskeleton marker, villin, revealed the expression of these proteins in XF-HIOs. Interestingly, the localization of villin indicated that our organoids were uniquely structured outwards and oriented toward the epithelial layers. Moreover, XF-HIOs expressed Muc2 as revealed by immunostaining and ELISA revealed Muc2 secretion into the supernatant (Figure 1C right panel, Figure S1A,B). From these results, we showed that XF-HIOs had a unique cell structure, similar to that found in previous experiments.

### 3.2. Nutrition-Related Transporters on Epithelial Cells

**Figure 2:**
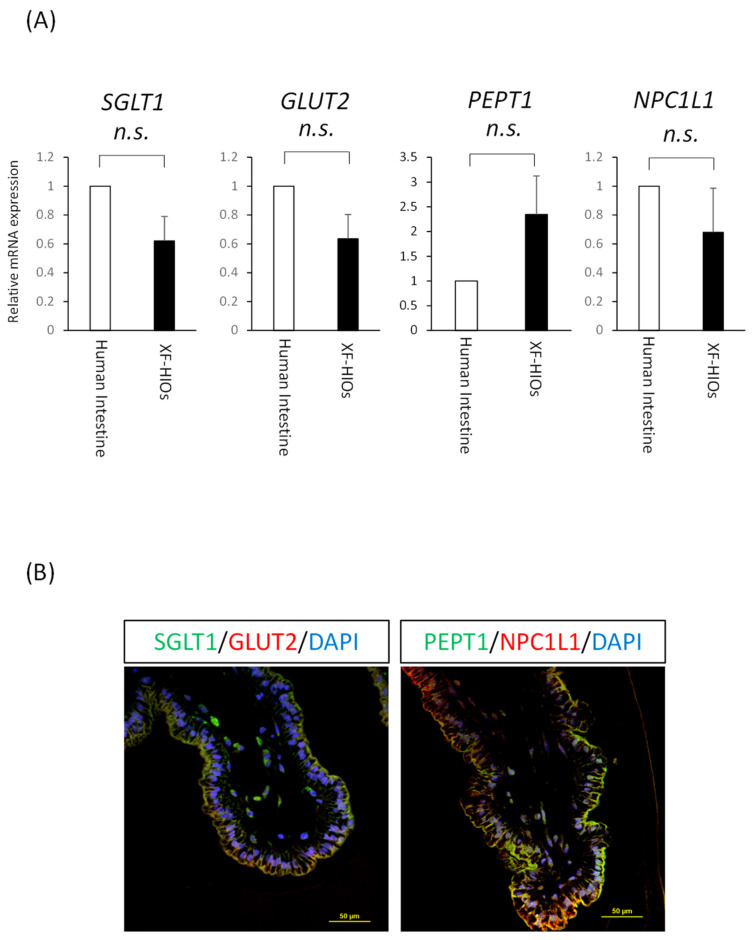
XF-HIOs possess nutrient-absorption-related transporters. (**A**) The level of expression of mRNAs for SGLT1, GLUT2, PEPT1, and NPC1L1 relative to the human small intestine. Data represent the mean ± S.E. values of three independent experiments. n.s., not significant. (**B**) Immunostaining images of three-dimensional xenogenic-free human intestinal organoids (XF-HIOs) with anti–Sodium/Glucose Cotransporter 1 (SGLT1), Glucose Transporter Type 2 (GLUT2), H+/oligopeptide cotransporter (PEPT1), and Niemann-Pick C-1–like-1 (NPC1L1) antibodies. Scale bars: 50 μm.

Next, we examined whether nutrient absorption could be analyzed using XF-HIOs. We focused specifically on three major nutrients: a sugar (glucose), protein (peptide), and fat (cholesterol), whose absorption is mainly mediated by transporters on enterocytes (Figure S2). To analyze the expression of nutrition-related transporter genes, mRNA analysis and immunostaining were undertaken. As shown in Figure 2A, the expression of mRNAs of glucose transporters, SGLT1 and GLUT2, peptide transporter 1, PEPT1, and cholesterol transporter, NPC1L in XF-HIOs, were identical to or elevated compared to those of the human small intestine. Moreover, the localization of proteins related to the transporters was in an outward direction alongside villin expression, suggesting XF-HIOs may have absorptional functions for each nutrient (Figure 2B). For comparison, immunostaining using human small intestine and a Caco-2 monolayer, a colon cancer cell line, was performed and showed transporter expression in the apical membrane (Figure S3A,B). These data suggest that nutrition-related transporters were expressed outwardly and might absorb nutrients from the external space of XF-HIOs even while maintaining a 3D form.

### 3.3. XF-HIOs Absorb Glucose, Dipeptide, and Cholesterol

**Figure 3:**
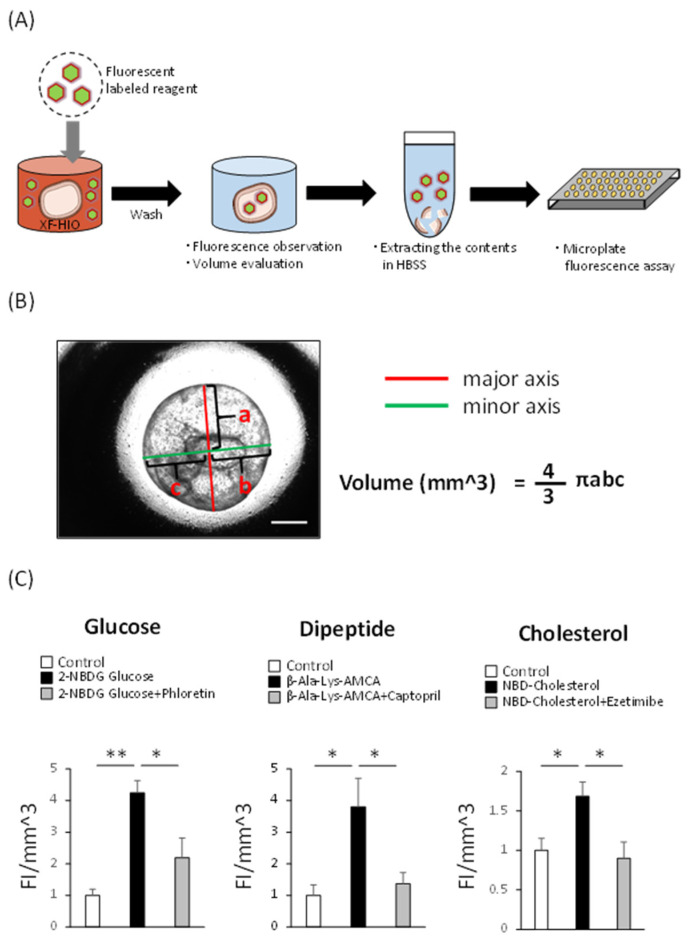
Absorption of glucose, dipeptide, and cholesterol into XF-HIOs in a transporter-dependent manner. (**A**) Diagram of the analysis of absorption using a fluorescent-labeled reagent. (**B**) The major and minor axes of a three-dimensional xenogeneic-free human intestinal organoid (XF-HIO) were automatically measured by the image analysis [19], and the volume was used for the correction of fluorescence intensity. Scale bar: 500 μm. (**C**) The levels of internal fluorescence intensity of labeled glucose, dipeptide, and cholesterol; control (open columns), treatment with fluorescent-labeled reagents (black columns) and with inhibitors, phloretin, captopril (1 mM) and ezetimibe (20 μM) (gray columns). PBS, phosphate-buffered saline; HBSS, Hank’s buffered saline solution. Data are presented as the mean ± S.E. values of four independent experiments. * *p* < 0.05 and ** *p* < 0.01.

Next, we estimated nutrient absorption by XF-HIOs. Our protocol for the assessment of absorption is illustrated in Figure 3A. Three-dimensional xenogeneic-free human intestinal organoids were exposed to each fluorescent-labeled reagent, homogenized in HBSS+, and their fluorescence intensity measured using a microplate reader. After measurement, values were re-calculated in terms of per organoid volume. The volume was calculated using a general formula for spheres or ellipsoids (Figure 3B) in our image-analyzing system. Before the analysis, we examined the barrier function of XF-HIOs according to a previously published protocol [20]. No significant difference in the permeation of FD-4 was observed between untreated and treated XF-HIOs (Figure S4A). Additionally, Figure S4B highlights the expression of the tight junction protein, zonula occludens-1, in XF-HIOs. These data suggest that XF-HIOs had sufficient epithelial barrier function for the absorption analysis. As a result, we quantified the amount of fluorescent-labeled glucose, dipeptide, and cholesterol, and examined the effect of their inhibitors. We showed glucose absorption into XF-HIOs was decreased by phloretin, an inhibitor of the SGLT and GLUT families. Similar results were obtained concerning the absorption of dipeptide and cholesterol, and the effect of the inhibitors, captopril for dipeptides and ezetimibe for cholesterol, respectively (Figure 3C). In addition, these absorptions were visibly different under fluorescence microscopy (Figure S5), and the absorptions of glucose and dipeptide showed dose dependency (Figure S6). A ten times higher concentration of the fluorescent-labeled cholesterol showed a significantly lower absorption rate, because a higher concentration of the substance could damage the tissue structure of the XF-HIOs. These results clearly demonstrated that XF-HIOs assimilated the three major nutrients examined through specific transporters.

### 3.4. Decreased Nutrient Absorption Caused by Food Composition

**Figure 4:**
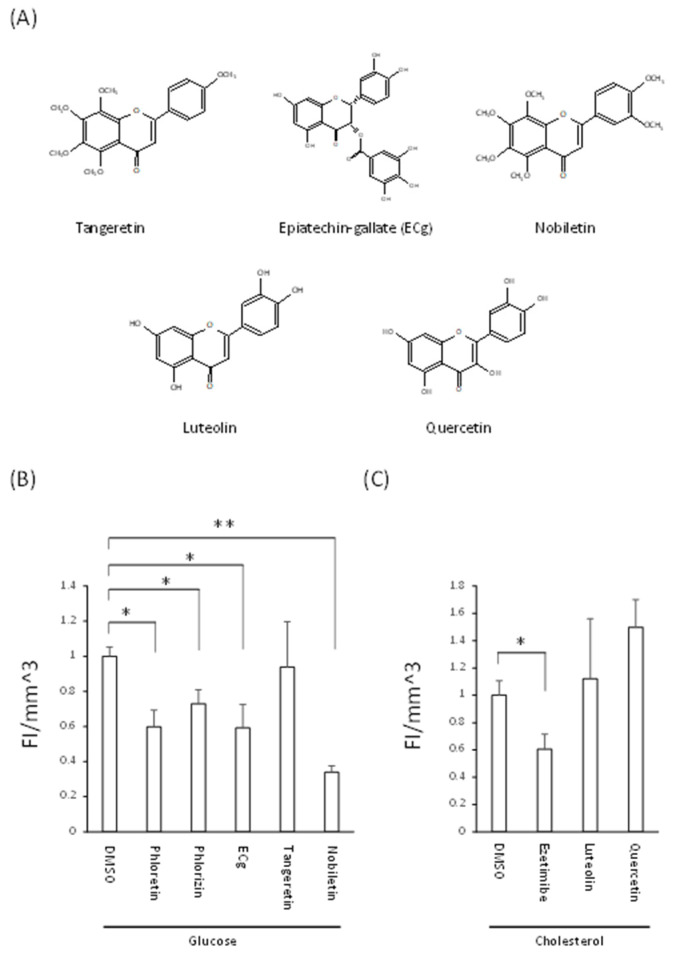
Inhibitory effect on glucose and cholesterol absorption. (**A**) Chemical structure of tangeretin, epicatechin-gallate, nobletin, luteolin, and quercetin. (**B**) Glucose absorption in three-dimensional xenogeneic-free human intestinal organoid (XF-HIOs) after treatment with phloretin, phlorizin (1 mM), tangeretin (25 μM), epicatechin-gallate (25 μM) or nobletin (25 μM). (**C**) Cholesterol absorption in XF-HIOs after treatment with ezetimibe (20 μM), luteolin (100 μM) or quercetin (100 μM). Data are presented as the mean ± S.E. of four independent experiments. * *p* < 0.05 and ** *p* < 0.01. DMSO, dimethyl sulfoxide.

Glucose and cholesterol are essential ingredients for homeostasis. However, their excessive ingestion causes diabetes and hyperlipemia, respectively [21,22]. Previous studies using Caco-2 cells or animal models showed that several different food compositions suppressed their absorption [23,24]. In this study, we studied fluorescent-labeled glucose and cholesterol absorption in assays of XF-HIOs using several different food compositions. Representative chemical agents are described in Figure 4A. Epicatechin-gallate, tangeretin, and nobletin suppressed glucose absorption [23], and luteolin and quercetin suppressed that of cholesterol [24]. In the case of XF-HIOs, for glucose, absorption was decreased by phloretin, phlorizin, ECg and nobletin, but not by tangeretin (Figure 4B). Epicatechin gallate was effective even at 1% concentrations under the same assay conditions (Figure S7). For cholesterol, luteolin and quercetin did not affect its absorption (Figure 4C). Taken together, this indicates that glucose absorption can be suppressed by taking foods containing natural ingredients as analyzed in XF-HIOs.

## 4. Discussion

We previously reported that our XF-HIOs generally showed in vivo intestinal properties, as revealed by cellular composition, peristaltic motion, and drug metabolism [20,23]. In this study, we newly analyzed the transporter-dependent absorption of nutrients in XF-HIOs. Several previous studies used human or murine-derived intestinal organoids, which showed functional nutrient absorption [25,26]. However, the brush border of these organoids is inward in structure and nutritional-absorption-related transporters are also present in the same direction. For the analysis of absorption, organoids must be separated into cells to expose the apical side of enterocytes. In contrast, XF-HIOs have an inverse structure, meaning that the brush border of intestinal epithelial cells as well as transporters are directed outward (Figure 1C and Figure 2B). Therefore, this suggests that XF-HIOs can be used as a tool to evaluate absorption without dissociation into cells (Figure 3A). Moreover, Caco-2 colorectal cancer cells are widely used as representative material for the analysis of transporters, but several groups have cast doubt on any similarity with normal human intestine [10,11].

In this study, except for PEPT1, transporter expression in XF-HIOs was statistically stable, showing little difference from that of the human intestine (Figure 2A). Glucose, dipeptide, and cholesterol absorption in XF-HIOs were subsequently shown, and the inhibitory effect that was dependent on each transporter clarified (Figure 3C). In comparison, the evaluation of fructose absorption [27] and diffusional absorption by micellization of cholesterol [28] was not achieved, and comprehensive absorption efficiency was not analyzed; these remain future issues to be investigated. Using fluorescent reagents in evaluations did not appear problematic, even if compared with absorption and/or permeation experiments in Caco-2 cells (Figure S8). It is likely that the expression level of transporters in Caco-2 cells differs from that in the small intestine, and that the barrier property is stronger than that of the in vivo organ itself [10,11]. Regarding the characteristic functions of XF-HIOs, we have previously reported that Caco-2 cells do not show certain functions, such as metabolism-related enzyme expression and metabolic activity [17]; we therefore developed an alternative assay system using XF-HIOs.

Polyphenols have grown in interest as functional food substances in modern nutrients [29]; however previous reports suggested that a variety of intestinal effects in the flavonoids depend on the chemical structures of their polyphenols [23,24,30]. It predicted that the methoxy group at position 8 of the A-ring of the flavonoid skeleton, which is commonly found only in tangeretin and nobiletin, could be essential for the inhibition of transporter activity and that the presence of the methoxy residue in the compound affects its inhibitory capacity [30]. The intestinal absorption of glucose and cholesterol was found to be suppressed by several food compositions contained in vegetables, citrus or herbs [23,24]. In this study, unlike tangeretin, ECg and nobiletin suppressed glucose absorption in XF-HIOs (Figure 4B). The compound ECg, found in green tea catechin, was observed to decrease glucose absorption by competitive inhibition against SGLT 1 [31]. Since we showed an inhibitory effect of phloretin and phlorizin, suggesting glucose absorption via SGLT1 and GLUT2 (Figure 3C and Figure 4B), respectively, ECg is considered to have a similar transporter-dependent inhibitory effect in XF-HIOs. In contrast, although nobiletin and tangeretin, found in citrus fruits, have similar chemical structures, tangeretin did not sufficiently inhibit absorption in this study (Figure 4A,B). A previous study described how tangeretin was not as effective as nobiletin in inhibiting SGLT2 expressed on kidney cells [32]. In addition, other reports stated that the effect of tangeretin was weaker than that of ECg or nobiletin in inhibiting fructose absorption, and that the inhibitory effect of glucose absorption differed among various catechins [33,34]. Hence, tangeretin may have a low affinity for transporters and this might depend on its concentration. Therefore, further studies are needed to establish better analytical conditions. It is known that the partial reabsorption of glucose also occurs through SGLT2 in the kidney, making this a target for the development of diabetic drugs [35,36]. However, an inhibitor of absorption from the small intestine has not yet been developed so that XF-HIOs might be beneficial as a screening tool in this respect. In comparison, cholesterol absorption was not suppressed by luteolin and quercetin, compounds found in several vegetables (Figure 4C). Previous reports described how these two ingredients suppressed cholesterol absorption by inhibiting NPC1L1, but suppression by luteolin occurred after treatment for 48 h, and that by quercetin after 24 h [24,37]. Therefore, it is necessary to examine the reaction time and concentration of ingredients for cholesterol absorption as well as that of glucose. The functions of other nutrients, such as vitamins and minerals, and the optimization of XF-HIOs for such components need to be further investigated in the future.

## 5. Conclusions

Three-dimensional xenogeneic-free human intestinal organoids assimilated nutrients, glucose, peptide, and cholesterol via transporters expressed on the cellular surface. The administration of epicatechin gallate and nobiletin suppressed glucose absorption, although tangeretin for glucose and luteolin and quercetin for cholesterol had no significant effect on function. These results suggest the XF-HIOs, in form of 3D structures, are similar to the human intestine and can be used to better elucidate the absorption of natural food ingredients.

## Data Availability

The data presented in this study are available on request from the corresponding author.

## Supplementary Materials

The following are available online at https://www.mdpi.com/article/10.3390/nu14030438/s1. Table S1: The sequence of primer pairs used in this study; Table S2: List of antibodies; Figure S1: Muc2 was secreted outward in XF-HIOs; Figure S2: The absorption pathway via transporters of the three major nutrients in the small intestine; Figure S3: Human small intestine and Caco-2 cells each express nutrient-related transporters; Figure S4: XF-HIOs show tight junctions and barrier functions; Figure S5: The absorption of nutrients in XF-HIOs observed by fluorescence imaging; Figure S6: Permeation and/or absorption of fluorescent-labeled nutrients in Caco-2 cells. Figure S7: Inhibitory effect on glucose absorption with inhibitors’ dose-dependency in XF-HIOs. Figure S8: Permeation and/or absorption of fluorescent–labeled nutrients in Caco-2 cells.
