# Supplementary material for: Xenogeneic-Free Human Intestinal Organoids for Assessing Intestinal Nutrient Absorption
Source: Nutrients. 2022 Jan 19;14(3):438. doi: 10.3390/nu14030438 (PMC8838315; doi:10.3390/nu14030438)
Supplement: Supplementary file 1 [file nutrients-14-00438-s001.zip › nutrients-1535299-supplementary.pptx]

## Slide 1
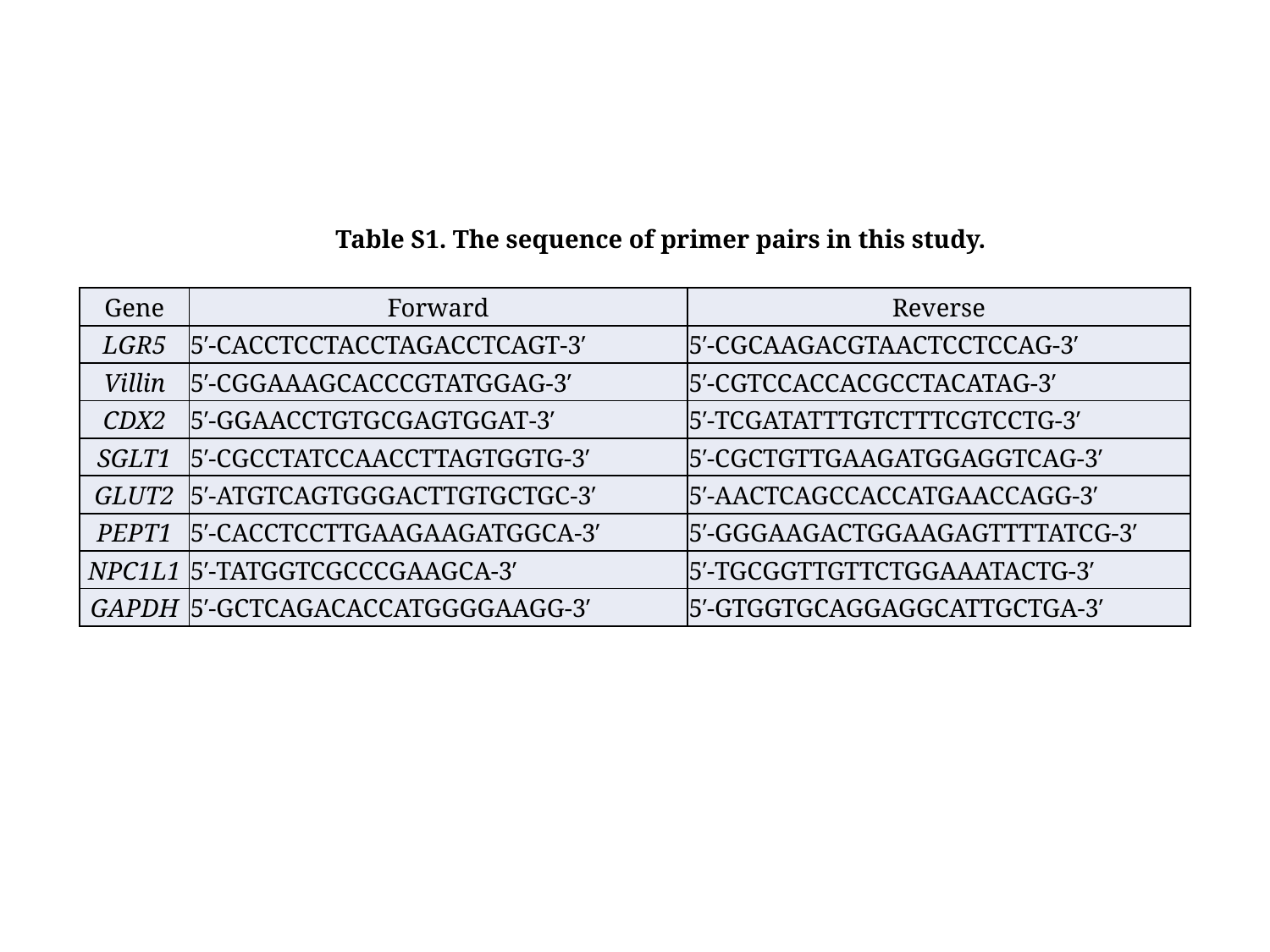

Table S1. The sequence of primer pairs in this study.
| Gene | Forward | Reverse |
| --- | --- | --- |
| LGR5 | 5′-CACCTCCTACCTAGACCTCAGT-3′ | 5′-CGCAAGACGTAACTCCTCCAG-3′ |
| Villin | 5′-CGGAAAGCACCCGTATGGAG-3′ | 5′-CGTCCACCACGCCTACATAG-3′ |
| CDX2 | 5′-GGAACCTGTGCGAGTGGAT-3′ | 5′-TCGATATTTGTCTTTCGTCCTG-3′ |
| SGLT1 | 5′-CGCCTATCCAACCTTAGTGGTG-3′ | 5′-CGCTGTTGAAGATGGAGGTCAG-3′ |
| GLUT2 | 5′-ATGTCAGTGGGACTTGTGCTGC-3′ | 5′-AACTCAGCCACCATGAACCAGG-3′ |
| PEPT1 | 5′-CACCTCCTTGAAGAAGATGGCA-3′ | 5′-GGGAAGACTGGAAGAGTTTTATCG-3′ |
| NPC1L1 | 5′-TATGGTCGCCCGAAGCA-3′ | 5′-TGCGGTTGTTCTGGAAATACTG-3′ |
| GAPDH | 5′-GCTCAGACACCATGGGGAAGG-3′ | 5′-GTGGTGCAGGAGGCATTGCTGA-3′ |

## Slide 2
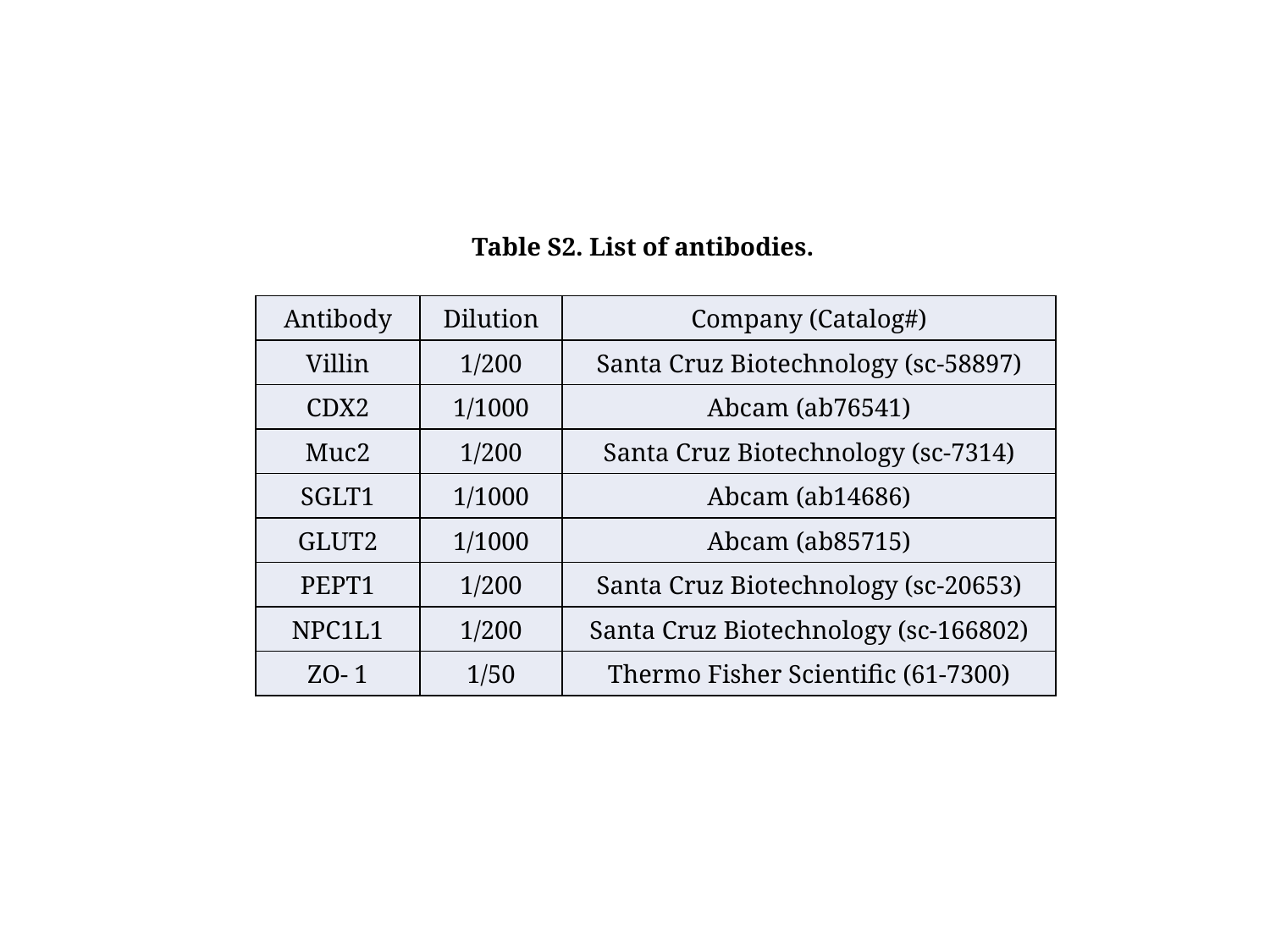

Table S2. List of antibodies.
| Antibody | Dilution | Company (Catalog#) |
| --- | --- | --- |
| Villin | 1/200 | Santa Cruz Biotechnology (sc-58897) |
| CDX2 | 1/1000 | Abcam (ab76541) |
| Muc2 | 1/200 | Santa Cruz Biotechnology (sc-7314) |
| SGLT1 | 1/1000 | Abcam (ab14686) |
| GLUT2 | 1/1000 | Abcam (ab85715) |
| PEPT1 | 1/200 | Santa Cruz Biotechnology (sc-20653) |
| NPC1L1 | 1/200 | Santa Cruz Biotechnology (sc-166802) |
| ZO- 1 | 1/50 | Thermo Fisher Scientific (61-7300) |

## Slide 3
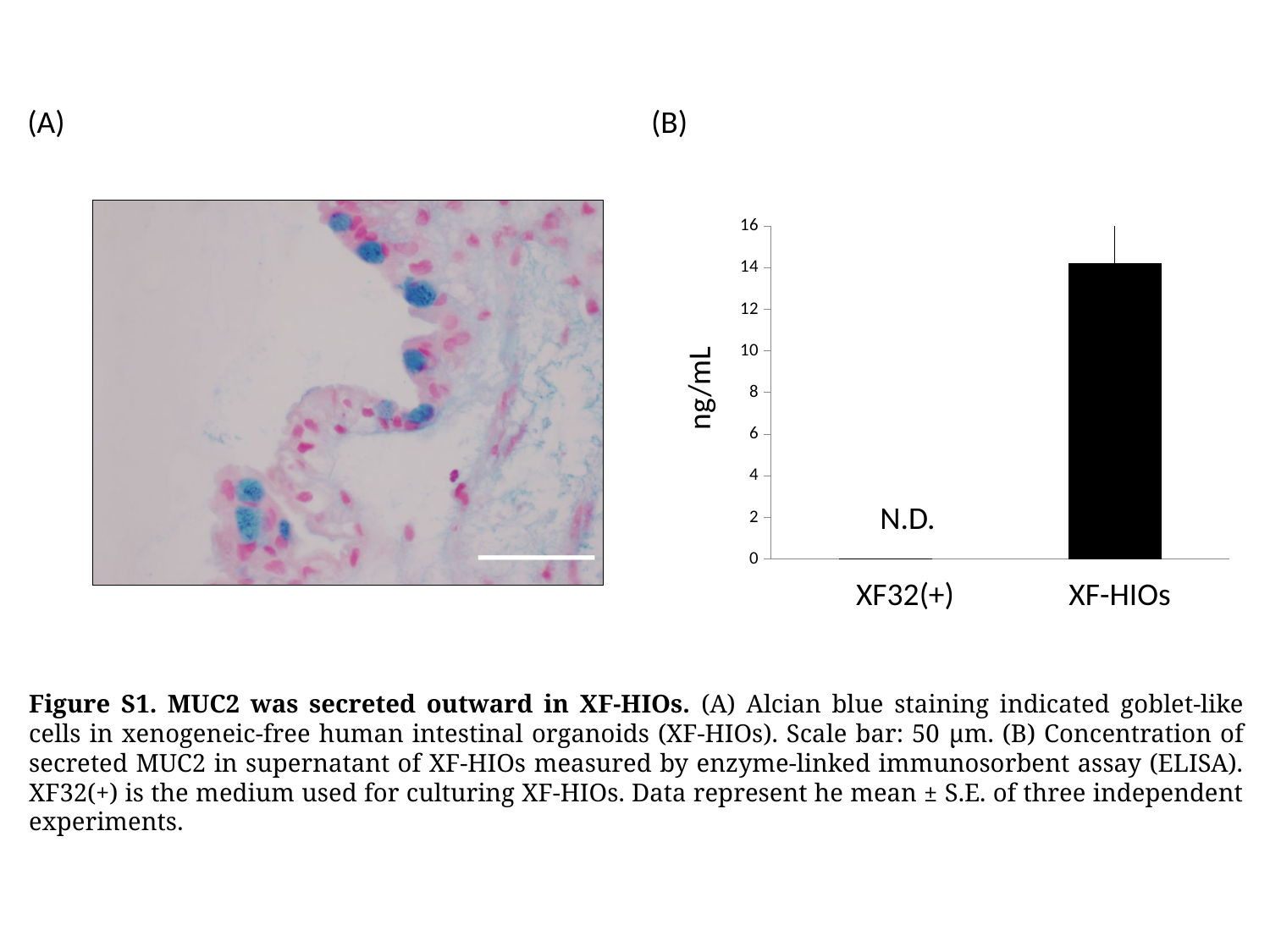

(A)
(B)
### Chart
| Category | |
|---|---|ng/mL
N.D.
XF32(+)
XF-HIOs
Figure S1. MUC2 was secreted outward in XF-HIOs. (A) Alcian blue staining indicated goblet-like cells in xenogeneic-free human intestinal organoids (XF-HIOs). Scale bar: 50 μm. (B) Concentration of secreted MUC2 in supernatant of XF-HIOs measured by enzyme-linked immunosorbent assay (ELISA). XF32(+) is the medium used for culturing XF-HIOs. Data represent he mean ± S.E. of three independent experiments.

## Slide 4
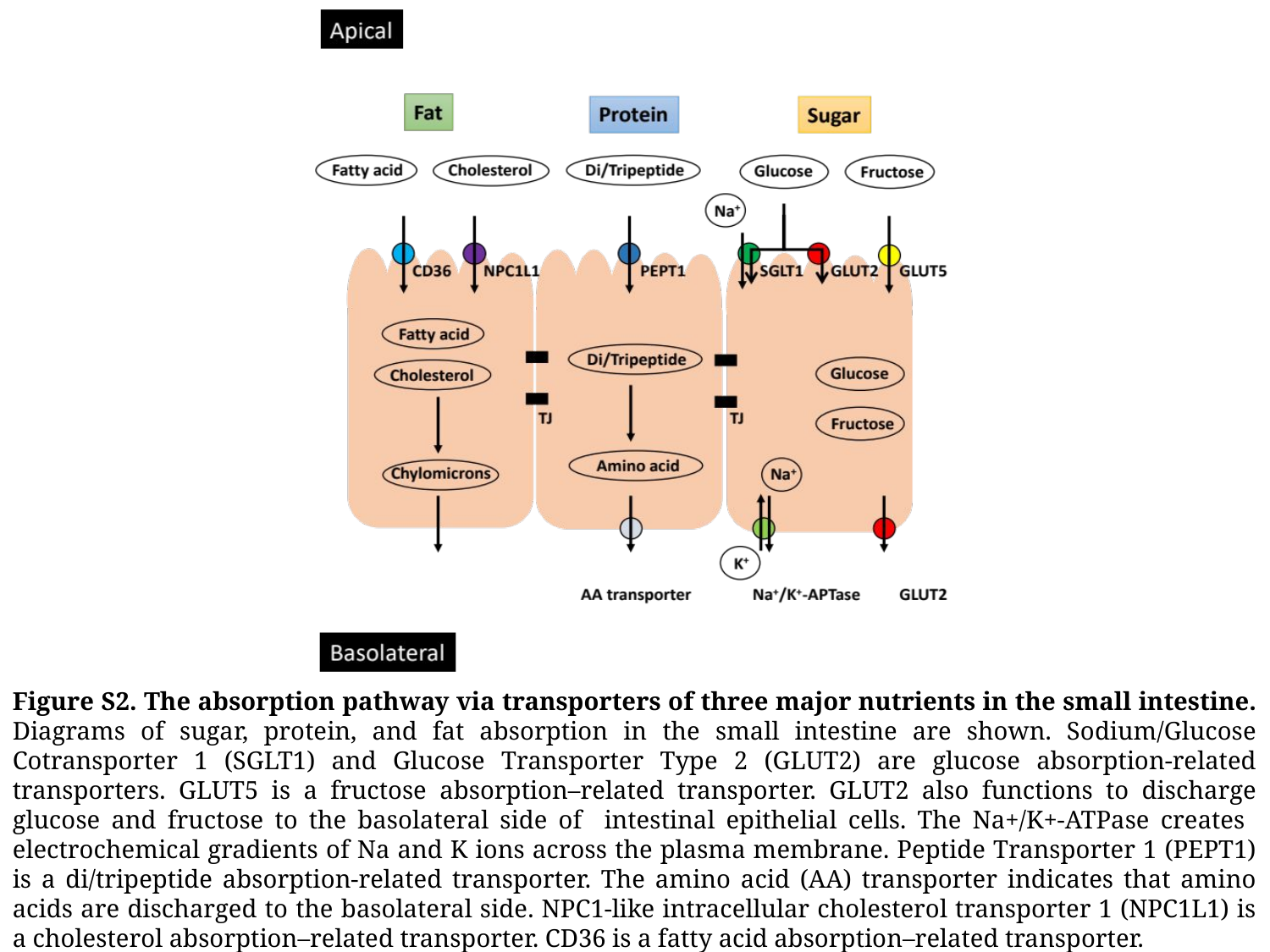

Figure S2. The absorption pathway via transporters of three major nutrients in the small intestine. Diagrams of sugar, protein, and fat absorption in the small intestine are shown. Sodium/Glucose Cotransporter 1 (SGLT1) and Glucose Transporter Type 2 (GLUT2) are glucose absorption-related transporters. GLUT5 is a fructose absorption–related transporter. GLUT2 also functions to discharge glucose and fructose to the basolateral side of intestinal epithelial cells. The Na+/K+-ATPase creates electrochemical gradients of Na and K ions across the plasma membrane. Peptide Transporter 1 (PEPT1) is a di/tripeptide absorption-related transporter. The amino acid (AA) transporter indicates that amino acids are discharged to the basolateral side. NPC1-like intracellular cholesterol transporter 1 (NPC1L1) is a cholesterol absorption–related transporter. CD36 is a fatty acid absorption–related transporter.

## Slide 5
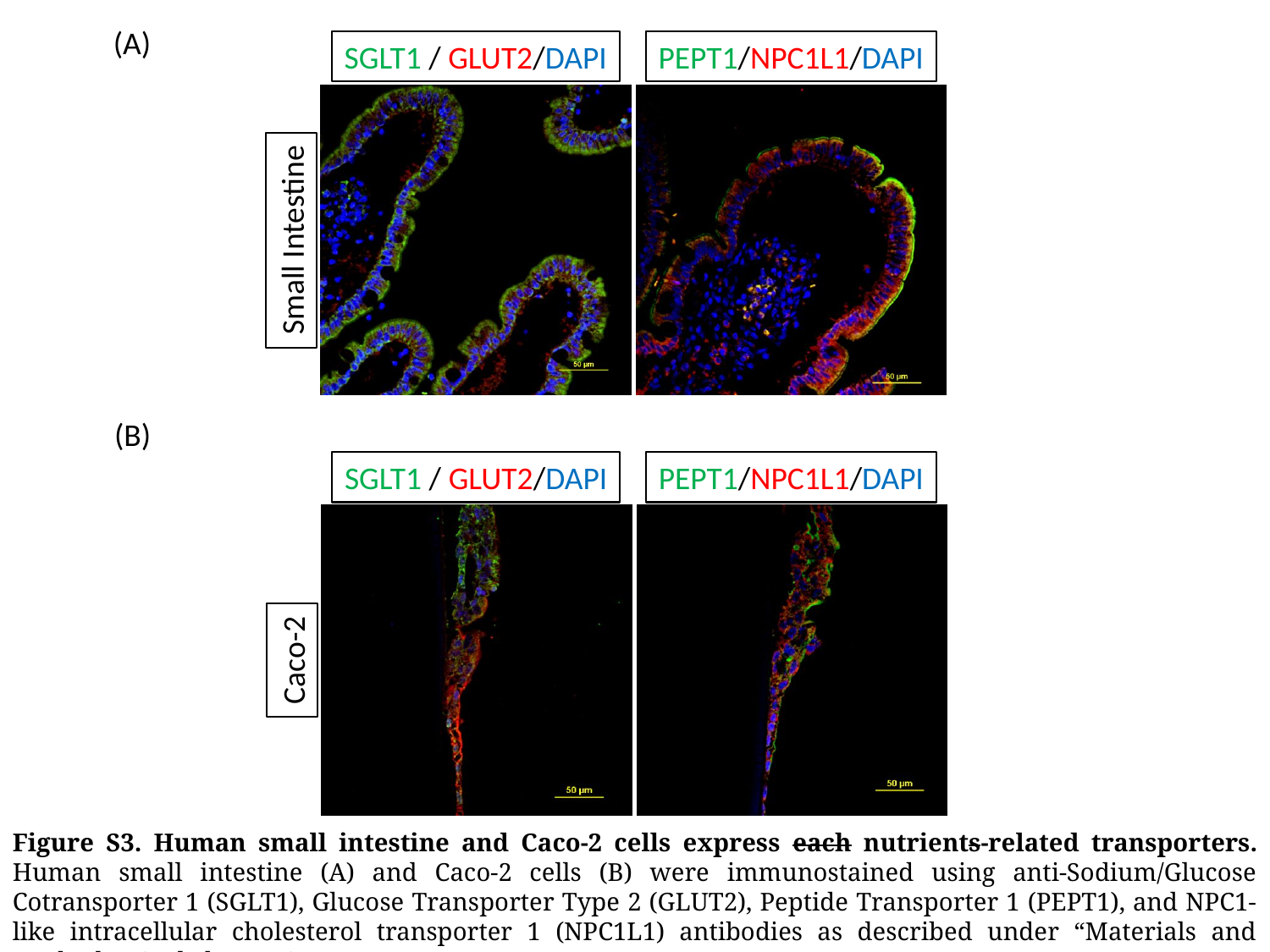

(A)
SGLT1 / GLUT2/DAPI
PEPT1/NPC1L1/DAPI
Small Intestine
PEPT1/NPC1L1/DAPI
SGLT1 / GLUT2/DAPI
Caco-2
(B)
Figure S3. Human small intestine and Caco-2 cells express each nutrients-related transporters. Human small intestine (A) and Caco-2 cells (B) were immunostained using anti-Sodium/Glucose Cotransporter 1 (SGLT1), Glucose Transporter Type 2 (GLUT2), Peptide Transporter 1 (PEPT1), and NPC1-like intracellular cholesterol transporter 1 (NPC1L1) antibodies as described under “Materials and methods.” Scale bars: 50 μm.

## Slide 6
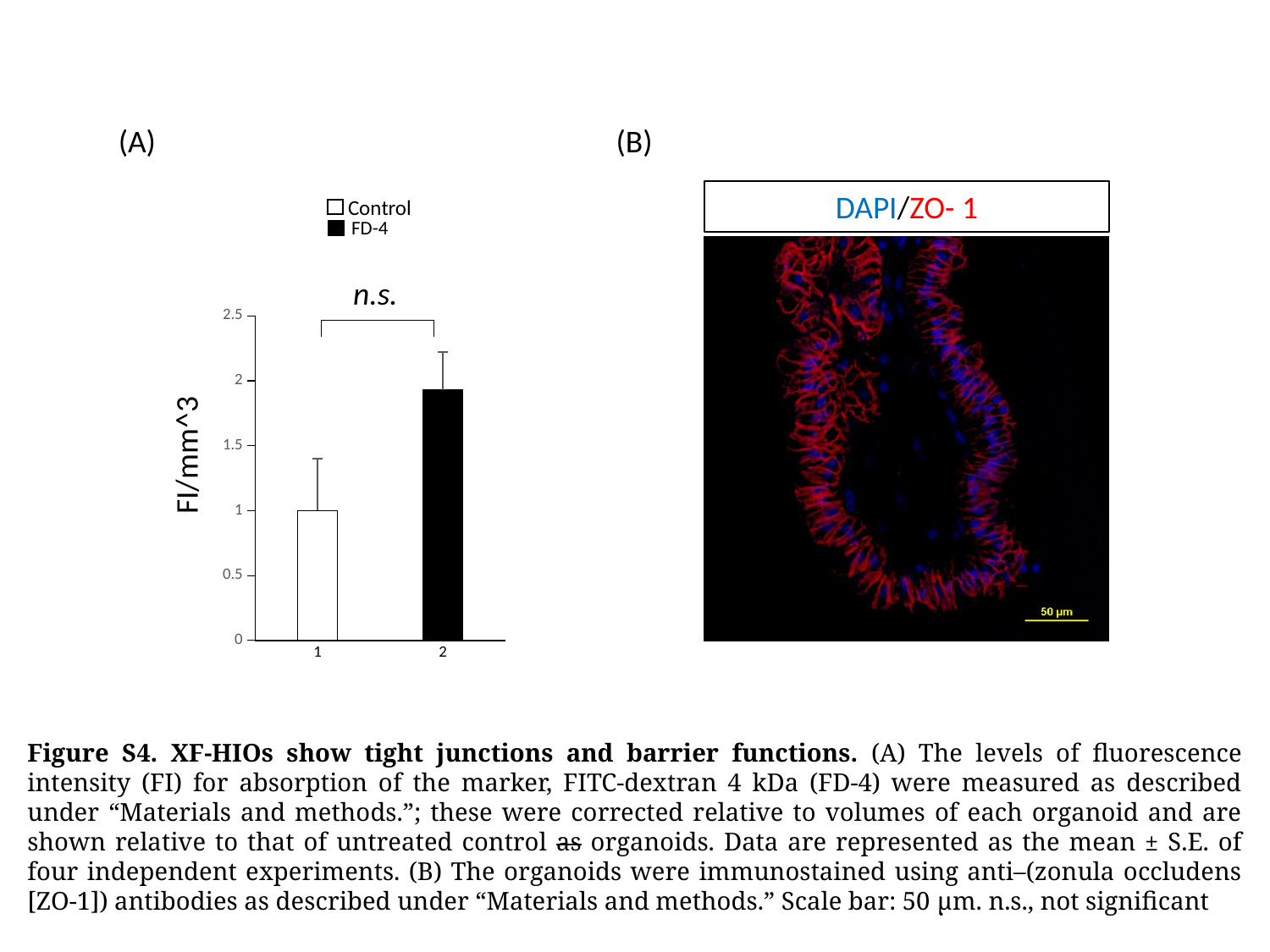

(A)
(B)
DAPI/ZO- 1
Control
FD-4
n.s.
### Chart
| Category | |
|---|---|FI/mm^3
Figure S4. XF-HIOs show tight junctions and barrier functions. (A) The levels of fluorescence intensity (FI) for absorption of the marker, FITC-dextran 4 kDa (FD-4) were measured as described under “Materials and methods.”; these were corrected relative to volumes of each organoid and are shown relative to that of untreated control as organoids. Data are represented as the mean ± S.E. of four independent experiments. (B) The organoids were immunostained using anti–(zonula occludens [ZO-1]) antibodies as described under “Materials and methods.” Scale bar: 50 μm. n.s., not significant

## Slide 7
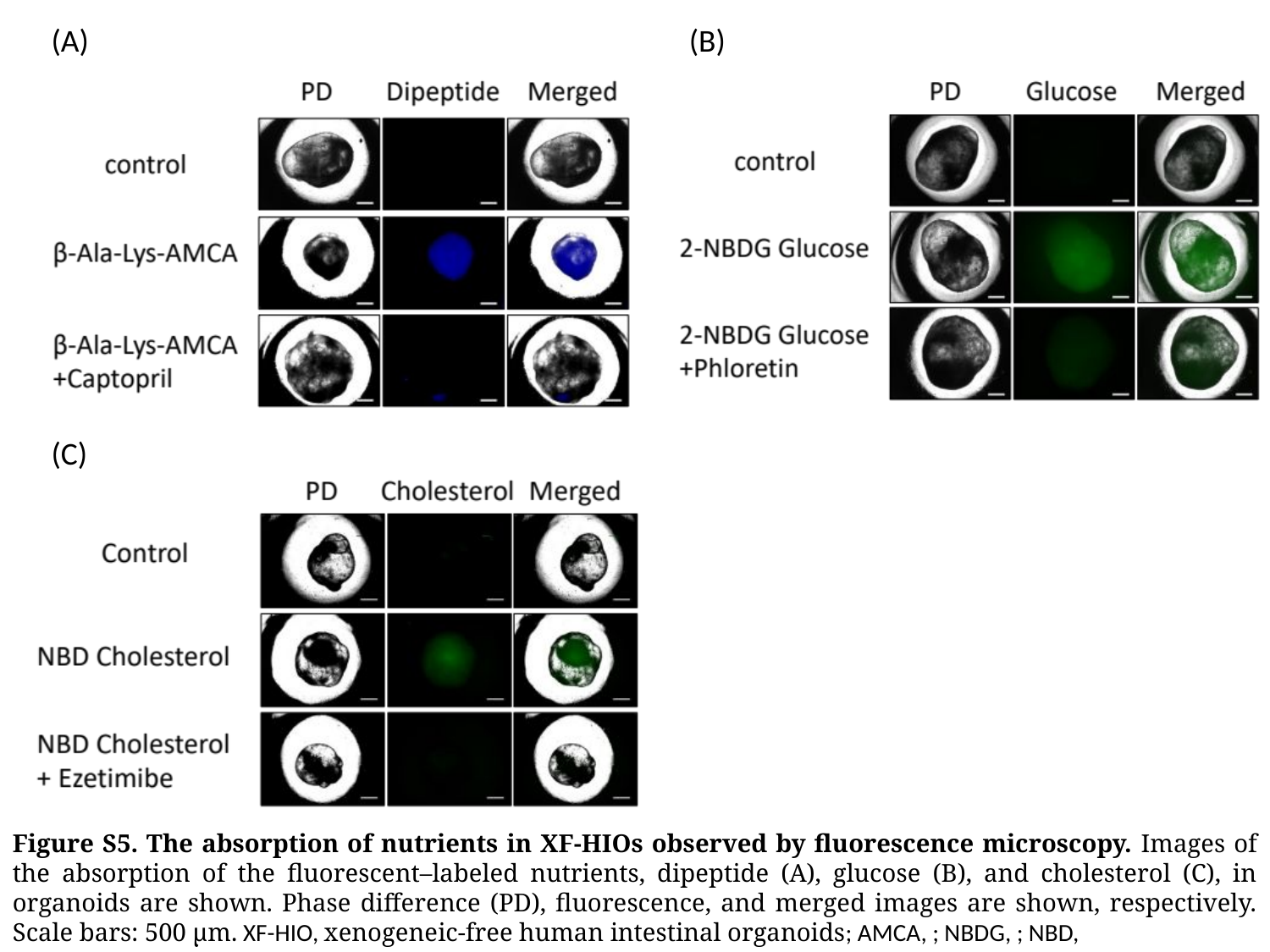

(A)
(B)
(C)
Figure S5. The absorption of nutrients in XF-HIOs observed by fluorescence microscopy. Images of the absorption of the fluorescent–labeled nutrients, dipeptide (A), glucose (B), and cholesterol (C), in organoids are shown. Phase difference (PD), fluorescence, and merged images are shown, respectively. Scale bars: 500 μm. XF-HIO, xenogeneic-free human intestinal organoids; AMCA, ; NBDG, ; NBD,

## Slide 8
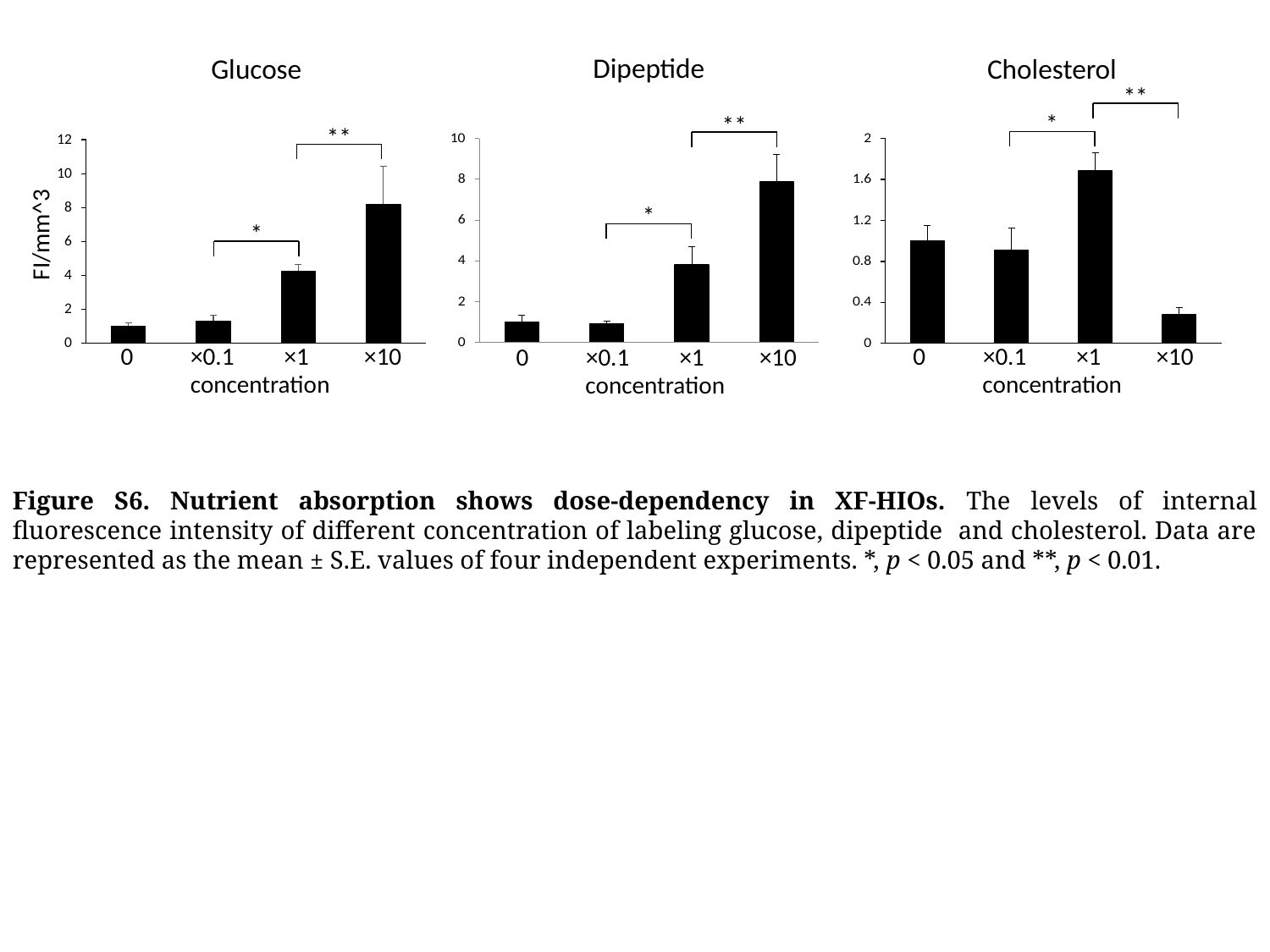

Dipeptide
Cholesterol
Glucose
**
*
**
**
*
*
FI/mm^3
0
×0.1
×1
×10
concentration
0
×0.1
×1
×10
concentration
0
×0.1
×1
×10
concentration
Figure S6. Nutrient absorption shows dose-dependency in XF-HIOs. The levels of internal fluorescence intensity of different concentration of labeling glucose, dipeptide and cholesterol. Data are represented as the mean ± S.E. values of four independent experiments. *, p < 0.05 and **, p < 0.01.

## Slide 9
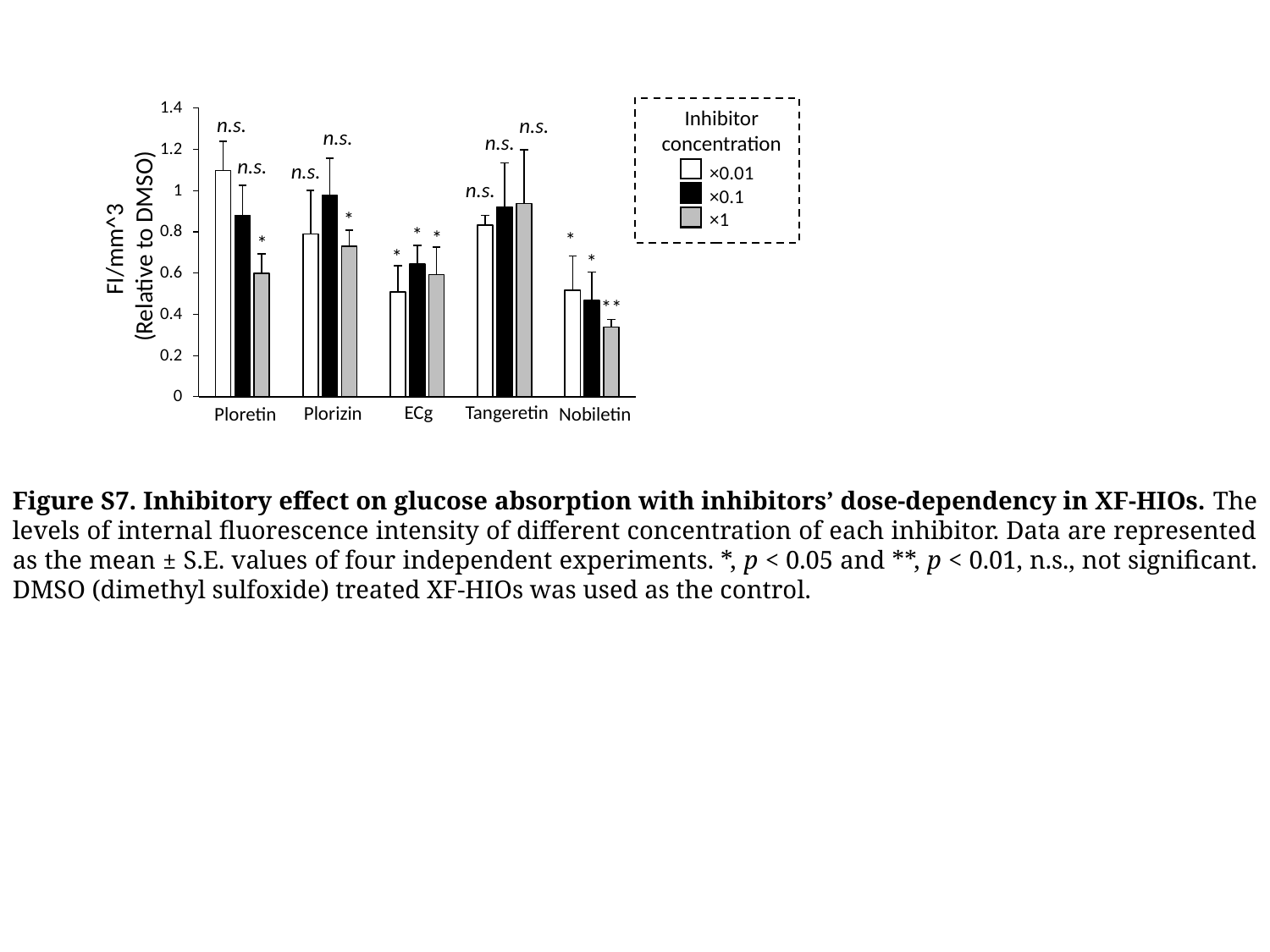

Inhibitor concentration
n.s.
n.s.
n.s.
n.s.
n.s.
n.s.
×0.01
×0.1
×1
n.s.
*
FI/mm^3
(Relative to DMSO)
*
*
*
*
*
*
**
ECg
Tangeretin
Plorizin
Ploretin
Nobiletin
Figure S7. Inhibitory effect on glucose absorption with inhibitors’ dose-dependency in XF-HIOs. The levels of internal fluorescence intensity of different concentration of each inhibitor. Data are represented as the mean ± S.E. values of four independent experiments. *, p < 0.05 and **, p < 0.01, n.s., not significant. DMSO (dimethyl sulfoxide) treated XF-HIOs was used as the control.

## Slide 10
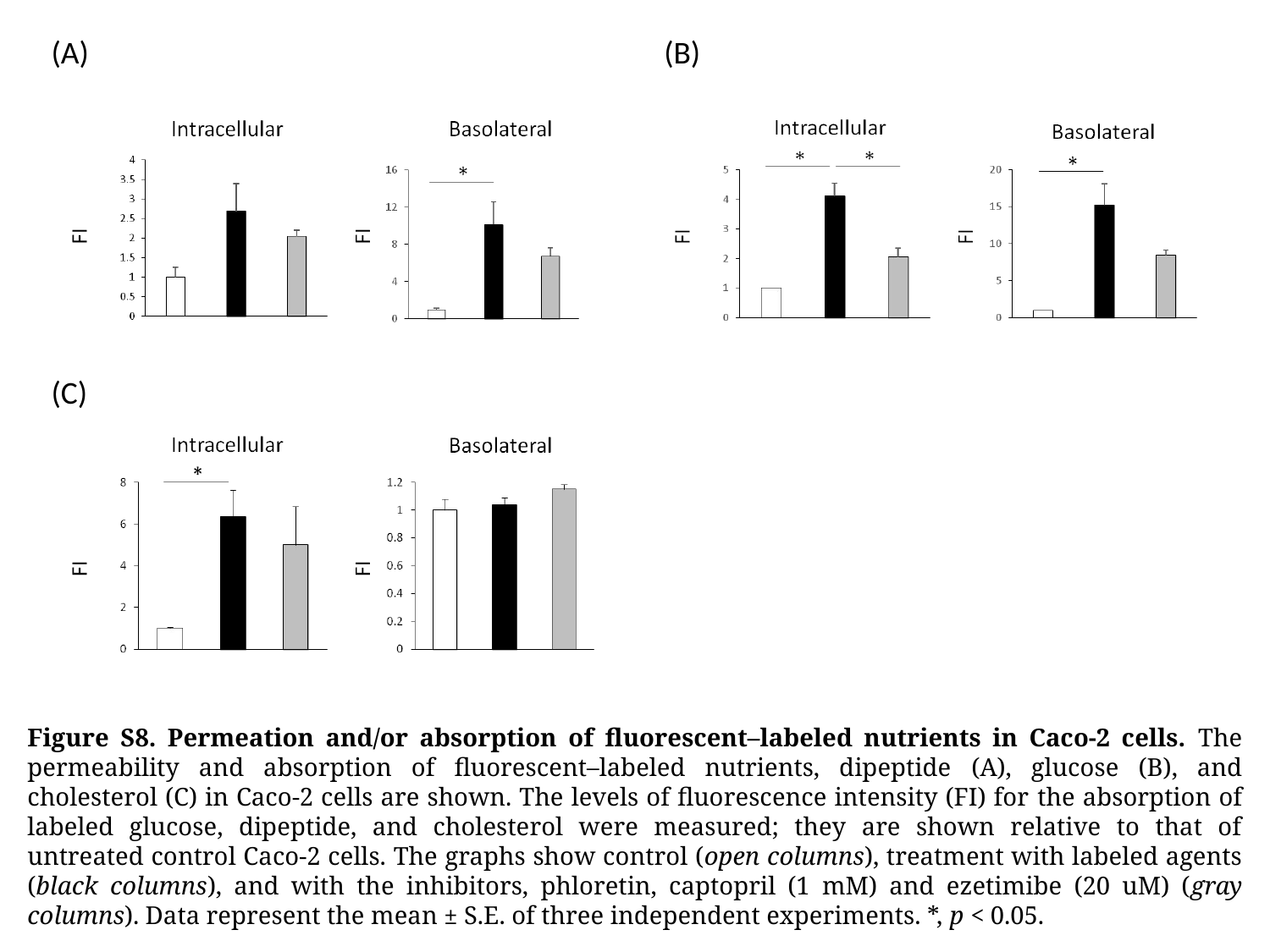

(A)
(B)
(C)
Figure S8. Permeation and/or absorption of fluorescent–labeled nutrients in Caco-2 cells. The permeability and absorption of fluorescent–labeled nutrients, dipeptide (A), glucose (B), and cholesterol (C) in Caco-2 cells are shown. The levels of fluorescence intensity (FI) for the absorption of labeled glucose, dipeptide, and cholesterol were measured; they are shown relative to that of untreated control Caco-2 cells. The graphs show control (open columns), treatment with labeled agents (black columns), and with the inhibitors, phloretin, captopril (1 mM) and ezetimibe (20 uM) (gray columns). Data represent the mean ± S.E. of three independent experiments. *, p < 0.05.
